# A potent Lassa virus antiviral targets an arenavirus virulence determinant

**DOI:** 10.1371/journal.ppat.1007439

**Published:** 2018-12-21

**Authors:** Ikenna G. Madu, Megan Files, Dima N. Gharaibeh, Amy L. Moore, Kie-Hoon Jung, Brian B. Gowen, Dongcheng Dai, Kevin F. Jones, Shanthakumar R. Tyavanagimatt, James R. Burgeson, Marcus J. Korth, Kristin M. Bedard, Shawn P. Iadonato, Sean M. Amberg

**Affiliations:** 1 Kineta, Inc., Seattle, Washington, United States of America; 2 SIGA Technologies, Inc., Corvallis, Oregon, United States of America; 3 Institute for Antiviral Research, Department of Animal, Dairy and Veterinary Sciences, Utah State University, Logan, Utah, United States of America; Division of Clinical Research, UNITED STATES

## Abstract

Arenaviruses are a significant cause of hemorrhagic fever, an often-fatal disease for which there is no approved antiviral therapy. Lassa fever in particular generates high morbidity and mortality in West Africa, where the disease is endemic, and a recent outbreak in Nigeria was larger and more geographically diverse than usual. We are developing LHF-535, a small-molecule viral entry inhibitor that targets the arenavirus envelope glycoprotein, as a therapeutic candidate for Lassa fever and other hemorrhagic fevers of arenavirus origin. Using a lentiviral pseudotype infectivity assay, we determined that LHF-535 had sub-nanomolar potency against the viral envelope glycoproteins from all Lassa virus lineages, with the exception of the glycoprotein from the LP strain from lineage I, which was 100-fold less sensitive than that of other strains. This reduced sensitivity was mediated by a unique amino acid substitution, V434I, in the transmembrane domain of the envelope glycoprotein GP2 subunit. This position corresponds to the attenuation determinant of Candid#1, a live-attenuated Junín virus vaccine strain used to prevent Argentine hemorrhagic fever. Using a virus-yield reduction assay, we determined that LHF-535 potently inhibited Junín virus, but not Candid#1, and the Candid#1 attenuation determinant, F427I, regulated this difference in sensitivity. We also demonstrated that a daily oral dose of LHF-535 at 10 mg/kg protected mice from a lethal dose of Tacaribe virus. Serial passage of Tacaribe virus in LHF-535-treated Vero cells yielded viruses that were resistant to LHF-535, and the majority of drug-resistant viruses exhibited attenuated pathogenesis. These findings provide a framework for the clinical development of LHF-535 as a broad-spectrum inhibitor of arenavirus entry and provide an important context for monitoring the emergence of drug-resistant viruses.

## Author summary

Lassa fever is a viral hemorrhagic fever disease that is transmitted to humans primarily through contact with the urine or feces of infected rodents. The disease is endemic in West Africa, and an unusually large outbreak occurred in Nigeria in early 2018. The case fatality rate was 25% among confirmed cases, underscoring the need for an effective antiviral therapy. Here, we evaluated the small-molecule drug LHF-535, which targets the arenavirus envelope glycoprotein, for broad-spectrum activity against Lassa viruses of different lineages and related arenaviruses that cause hemorrhagic fever diseases in South America. We also selected for LHF-535-resistant viruses and characterized their genotype and phenotype. Using a combination of surrogate systems and wild-type viruses, we determined that all tested Lassa virus strains and New World hemorrhagic fever arenaviruses were sensitive to LHF-535. Sensitivity to the drug was modulated by specific amino acid changes in the viral envelope glycoprotein, and the majority of emerging drug-resistant viruses were attenuated for virulence. Similarly, the live-attenuated vaccine strain for Argentine hemorrhagic fever was also resistant to LHF-535. These findings indicate that LHF-535 targets a viral virulence determinant, the mutation of which may result in the emergence of drug-resistant viruses, but with reduced capacity for virulence.

## Introduction

Targeting virulence factors is an intriguing antimicrobial strategy that may reduce selective pressure and delay or prevent emergence of antimicrobial resistance [1], but this approach has not been appreciably explored as an antiviral strategy. Antiviral drugs typically suppress viral replication and decrease viral loads, which can drive selection of less-sensitive variants and ultimately reduction in therapeutic effectiveness. We provide here an example of an antiviral that suppresses viral replication in a manner regulated by amino acids within the viral envelope glycoprotein that also contribute to virulence. The ramifications of this are a successful live vaccine strain that exhibits antiviral resistance and a tendency for drug-resistant variants to exhibit attenuation.

Lassa fever is a viral hemorrhagic fever (VHF), a serious illness characterized by bleeding diathesis, fever, multiple organ involvement, and high fatality rate. Because of the high prevalence of Lassa fever and the lack of approved therapeutic options or a protective vaccine, a World Health Organization panel of public health experts and scientists recently identified Lassa fever as one of eight disease priorities needing urgent research and development attention [2]. Lassa fever, one of the most frequently occurring VHFs, is caused by Lassa virus, a member of the family *Arenaviridae* [3]. This family of enveloped RNA viruses is composed of snake (genera *Reptarenavirus* and *Hartmanivirus*) and rodent (genus *Mammarenavirus*) lineages [4, 5]; the rodent lineage is subdivided into Old World and New World viruses. Lassa virus is an Old World arenavirus, but several New World arenaviruses can cause VHF as well. Lassa virus is endemic in West Africa; estimates of several hundred thousand infections annually are widely cited [6, 7], although the true incidence of disease is unclear. There are four Lassa virus lineages with significant genetic diversity [8, 9], although it has been proposed that a fifth lineage could be defined by subdividing lineage IV [10].

Our research efforts are focused on LHF-535, a small-molecule antiviral under development for the treatment and prevention of Lassa fever and other hemorrhagic fevers such as Argentine (Junín virus) and Bolivian (Machupo virus) hemorrhagic fevers. The safety and pharmacokinetics of LHF-535 in humans is currently under evaluation in a Phase 1a trial. LHF-535 is an optimized analog of the benzimidazole derivative ST-193, a viral entry inhibitor that targets the arenavirus envelope glycoprotein (GP) [11–14]. ST-193 has demonstrated protective efficacy in a guinea pig model of lethal Lassa virus infection [11], and LHF-535 mediated 100% survival when dosing was initiated one or three days post challenge in this model (K.A. Cashman, personal communication). The arenavirus GP is proteolytically processed into three subunits that include a stable signal peptide (SSP), a receptor-binding subunit (GP1), and a transmembrane fusion subunit (GP2). Upon binding to a cell surface receptor, the virus is endocytosed and GP2 mediates fusion of the viral and endosomal membranes. Entry inhibitors such as ST-193 and LHF-535 are thought to bind to and stabilize a pre-fusion structure, thereby suppressing the conformational rearrangement of GP2 that is necessary for membrane fusion [15, 16].

We report here that all Lassa virus strains are sensitive to LHF-535; however, sensitivity of the lineage I LP strain was atypical, exhibiting a reduction in sensitivity that we determined was due to a single amino acid substitution in the GP transmembrane domain. Interestingly, this residue is located in the same position as the attenuation determinant of the Junín virus vaccine strain, Candid#1 [17]. Additionally, we demonstrate that Junín and Candid#1 viruses display differential sensitivity to LHF-535 in a manner dependent upon the attenuation determinant. Finally, we evaluated LHF-535 efficacy and sensitivity using Tacaribe virus, a New World arenavirus, and we show that emergent LHF-535-resistant viruses are often attenuated. These results could have important implications for the clinical use of LHF-535, both with respect to co-administration of vaccine and antiviral and for concerns regarding the evolution of drug resistance.

## Results

### LHF-535 exhibits potent antiviral activity against a broad array of hemorrhagic fever arenaviruses

**Fig 1:**
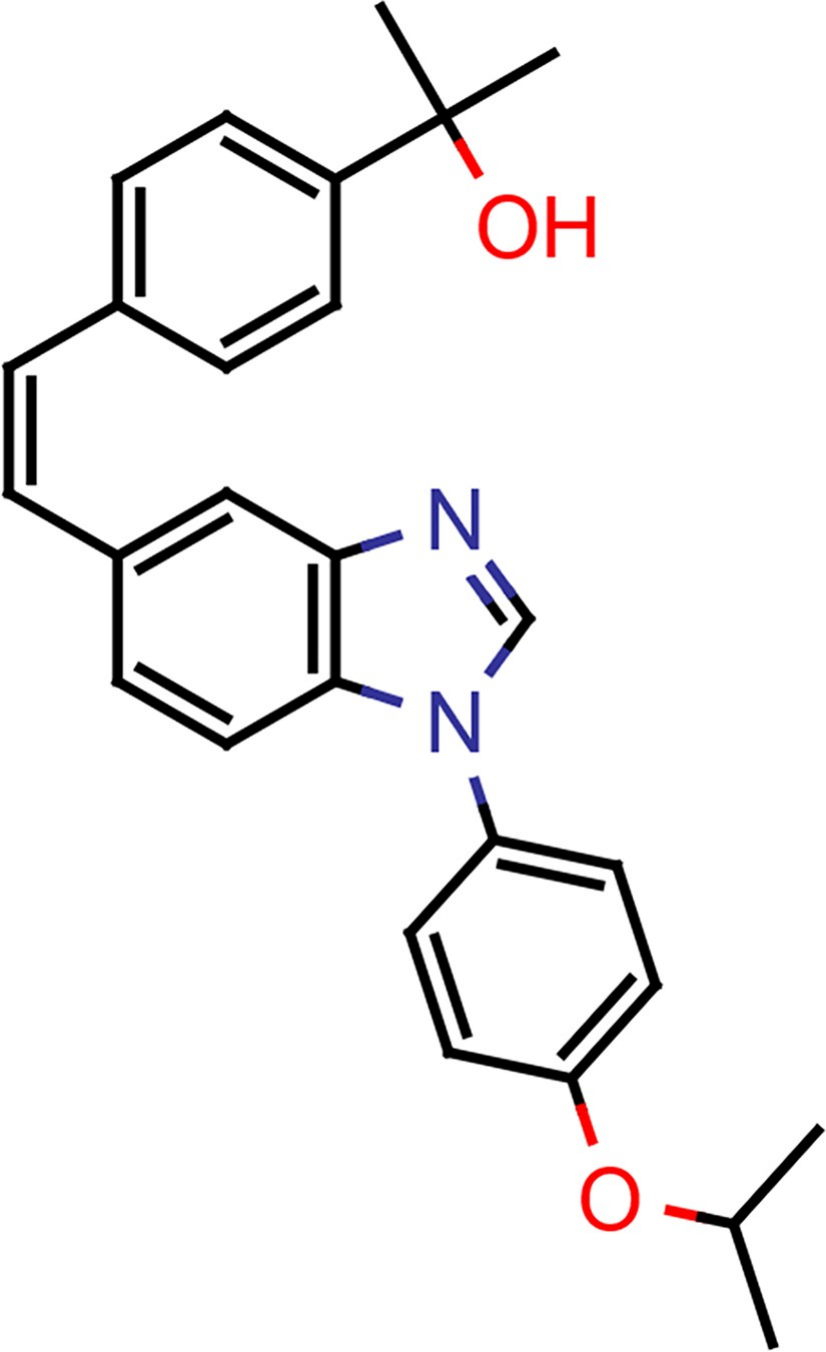
LHF-535 is a small-molecule compound of the bis-substituted benzimidazole class.

LHF-535, a chemical analog of the previously characterized arenavirus antiviral ST-193, was identified in a medicinal chemistry campaign to optimize the scaffold for advanced development as a therapeutic candidate (Fig 1). Lentiviral pseudotypes containing heterologous envelope GPs [12] were used to assess LHF-535-mediated inhibition of viral entry. Nine distinct Lassa virus strains were evaluated, including representatives from each of the four lineages (Table 1). Viral sensitivity to ST-193 was evaluated for reference. With the exception of the LP strain, LHF-535 inhibited Lassa GP-pseudotyped lentivirus with a 50% inhibitory concentration (IC_50_) of 0.1–0.3 nM. ST-193 was 2–6-fold less potent, with an IC_50_ of 0.4–1.4 nM. In contrast, the LP strain exhibited similar sensitivity to both ST-193 (12 nM) and LHF-535 (17 nM), but with an IC_50_ shift of 8–27 fold (ST-193) or 50–160 fold (LHF-535). Lentiviral pseudotypes expressing GPs from other Old World arenaviruses exhibited sensitivity to LHF-535 that was relative to their evolutionary distance from the Lassa virus lineages [18]. Inhibition of VSVg-pseudotyped lentivirus was used as a specificity control, and IC_50_ values against this virus generally mirrored cytotoxicity. IC_50_ values against lentiviral pseudotypes expressing GPs from LCMV or Lujo virus were similar to those against VSVg, suggesting little to no specific sensitivity of these viruses to either compound. New World arenavirus GPs exhibited a range of sensitivity (Table 1); the New World clade B viruses in particular were very sensitive, comparable to that of Lassa virus. Notably, the New World arenaviruses associated with hemorrhagic fever generally cluster in clade B [19].

**Table 1:** Sensitivity of envelope glycoproteins to viral entry inhibitors.

Envelope glycoprotein^a^					IC_50_ (μM)	
					ST-193	LHF-535
Old World arenaviruses	Lassa	Lineage I		LP	0.012	0.017
		Lineage II		803213	0.00055	0.00013
		Lineage III		CSF	0.00063	0.00033
				GA391	0.00044	0.00021
				Weller	0.00048	0.00018
		Lineage IV		AV^b^	0.00071	0.00013
				NL	0.0011	0.00023
				Z148	0.00058	0.00010
				Josiah	0.0014	0.00029
	Mobala				0.0040	0.00096
	Mopeia				0.0074	0.00081
	Gbagroube				0.00060	0.00029
	Menekre				0.35	0.12
		LCMV		Armstrong 53b	30	6.8
				Dandenong	18	5.8
	Lujo				15	3.9
New World arenaviruses	Clade A	Flexal			0.18	0.16
		Pichinde			2.6	2.7
	Clade B1	Junín			0.00065	0.00010
		Machupo		Carvallo	0.0030	0.00013
				Mallele	0.0014	0.000093
		Tacaribe			0.0042	0.00013
	Clade B2	Guanarito			0.00044	n.d.
	Clade B3	Chapare			0.058	0.019
		Sabiá			0.020	0.0020
Rhabdovirus	VSV				28	7.8

LCMV, lymphocytic choriomeningitis virus; n.d., not determined; VSV, vesicular stomatitis virus.

^a^Lentiviral pseudotypes incorporating heterologous envelopes were used to evaluate virus inhibition.

^b^Lassa virus AV strain has been suggested to be a member of a proposed lineage V [10].

### A single amino acid within GP2 modulates LHF-535 sensitivity of LP strain

**Fig 2:**
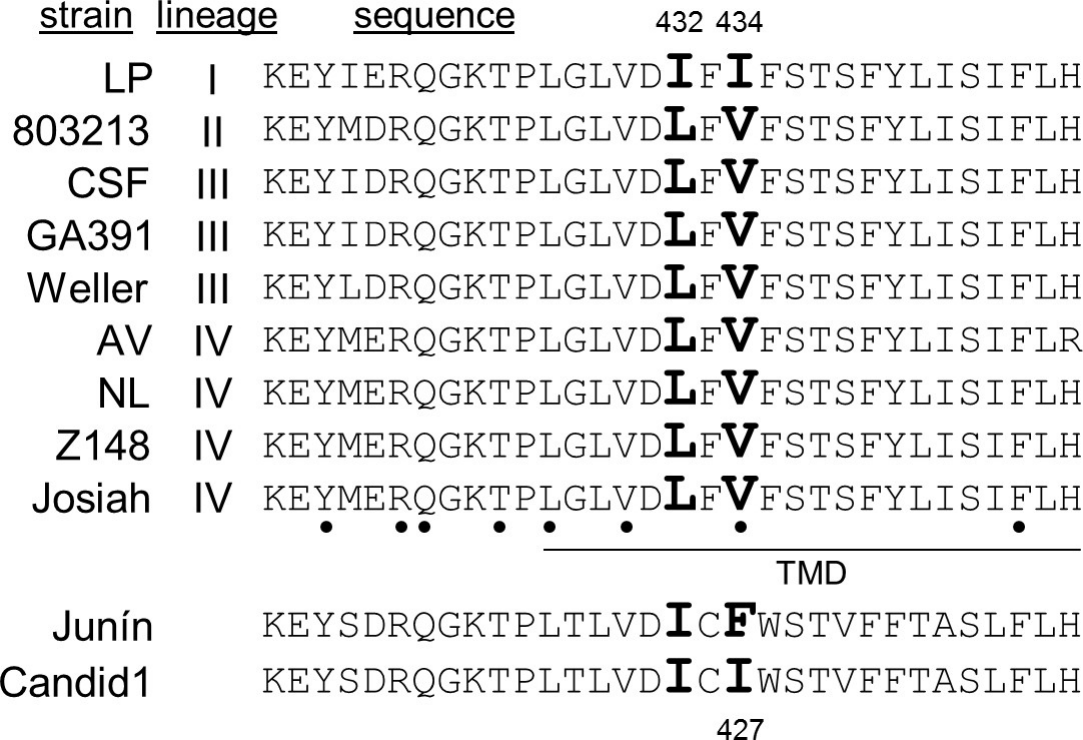
The Lassa virus LP strain contains two unique amino acid substitutions in the transmembrane domain of the GP2 subunit. The amino acid sequence shown is GP 417–448 of lineage IV and 416–447 for all others; previously identified sensitivity determinants are bulleted [12], the predicted transmembrane domain (TMD) is underlined, and the two distinguishing residues of the LP strain (I432 and I434) are in bold.

Compound sensitivity is regulated largely by amino acids in and near the predicted transmembrane domain of the GP2 subunit [12]. The transmembrane region is highly conserved across Lassa virus lineages, with two substitutions distinct to the LP strain (Fig 2). These two changes, I432 and I434, were independently replaced with the consensus residue, and the modified LP GPs were evaluated for LHF-535 sensitivity. LP I432L exhibited sensitivity similar to that of the parental LP GP, whereas LP I434V exhibited much higher sensitivity, equivalent to that of the other lineages (Table 2). The reciprocal substitution in the lineage IV background, Josiah V435I, exhibited reduced sensitivity to LHF-535, demonstrating that this position is responsible for modulating the divergent sensitivity of the LP strain (note that the corresponding amino acid numbering is offset by one due to an extra amino acid near the amino terminus of GP1 in lineage IV not found in the other lineages). This position of the transmembrane domain is also a ST-193 sensitivity determinant for Tacaribe virus, a New World arenavirus [12].

**Table 2:** LHF-535 sensitivity of engineered Lassa and Junín virus GPs.

Envelope GP	IC_50_ (μM)
Lassa LP	0.017
Lassa LP I432L	0.033
Lassa LP I434V	0.00012
Lassa Josiah	0.00029
Lassa Josiah V435I	0.025
Junín	0.00010
Junín F427I	> 20

### Junín virus sensitivity to LHF-535 is modulated by the Candid#1 attenuation determinant

Strikingly, Lassa virus GP V434 maps to an attenuation determinant of the Candid#1 live vaccine strain, which protects against Argentine hemorrhagic fever [20]. Candid#1 was derived by serial passage of Junín virus in guinea pigs, mice, and cultured rhesus monkey cells and contains multiple genetic changes; however, viral attenuation in mice [17] and guinea pigs [21] has been mapped to a F427I substitution in the GP2 subunit. The introduction of this attenuation determinant into the Junín virus GP (and incorporation into pseudotyped lentivirus) eliminated LHF-535 sensitivity (Table 2). To confirm this result with authentic replication-competent virus, virus yield reduction assays were run in parallel with Junín (Romero strain) and Candid#1 viruses. The sensitivity of the Romero strain to LHF-535 was several hundred-fold greater than that of the Candid#1 vaccine strain, whereas the two strains exhibited similar sensitivity to ribavirin (Table 3).

**Table 3:** LHF-535 exhibits differential potency in virus yield reduction assay.

Virus	LHF-535 IC_90_	ribavirin IC_90_
Junín (Romero)	0.0093 ± 0.0071 **μ**M (*n* = 3)	2.5 **μ**M (*n* = 1)
Junín (Candid#1)	3.0 ± 1.5 **μ**M (*n* = 3)	3.6 **μ**M (*n* = 1)

### LHF-535 protects mice in a lethal Tacaribe virus model

**Fig 3:**
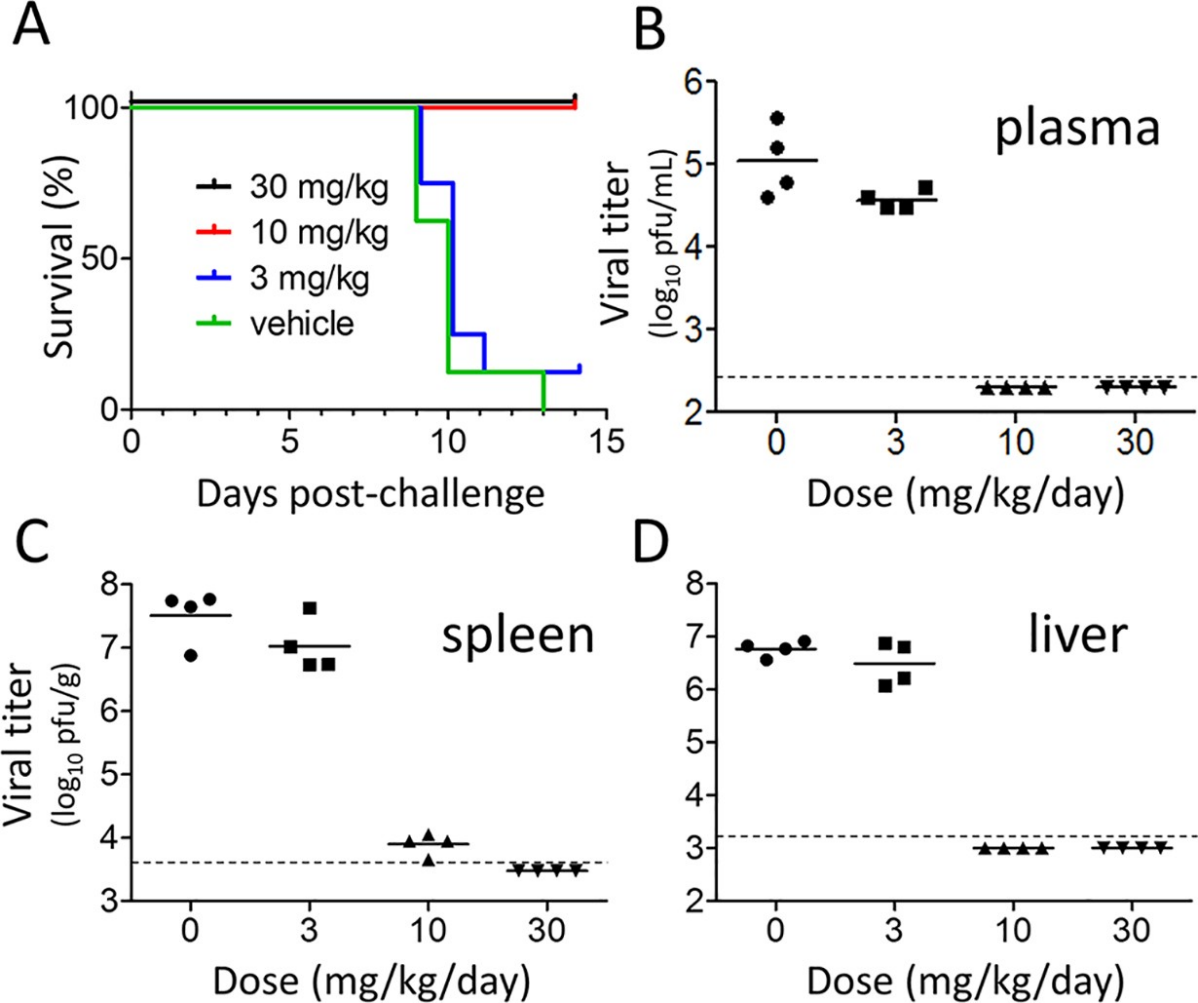
LHF-535 protects AG129 mice against lethal Tacaribe virus challenge. Mice were challenged with 200 plaque-forming units (PFU) of Tacaribe virus by intraperitoneal injection. Mice received daily oral doses of LHF-535 (3, 10, or 30 mg/kg) or vehicle alone beginning 0.5 h prior to challenge until 13 days post-challenge. One cohort (*n* = 8 animals per group) was monitored for survival (A), while another cohort (*n* = 4 animals per group) was sacrificed at 7 days post-challenge to measure viral titers in plasma (B), spleen (C), and liver (D). The virus detection limit is indicated by a dashed line.

**Fig 4:**
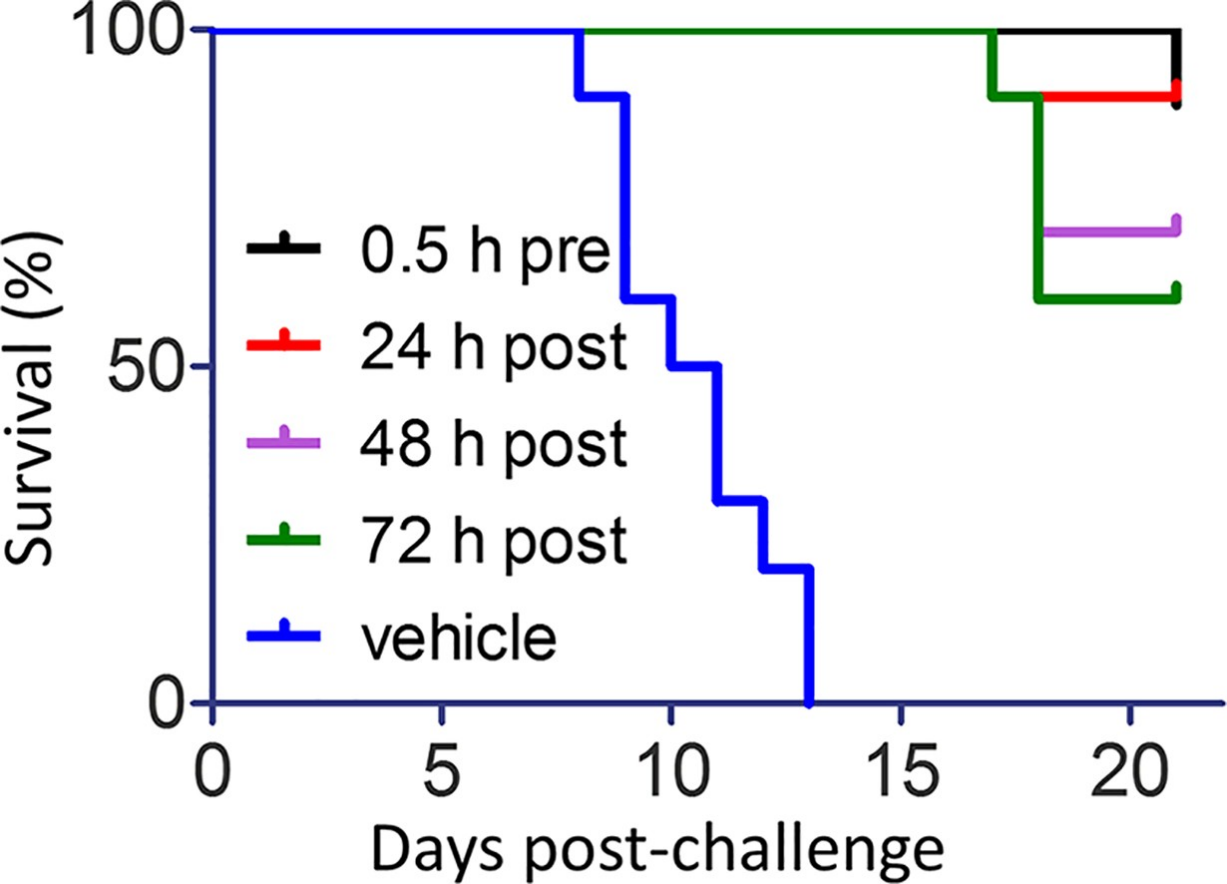
LHF-535 is effective as a post-exposure therapeutic in mice. AG129 mice (*n* = 10 per group) were challenged with Tacaribe virus as in Fig 3 and received daily oral doses of LHF-535 (10 mg/kg) beginning 0.5 h before, or 24, 48, or 72 h after viral challenge. All groups received their last dose at 13 days post-challenge. The control group was treated with vehicle alone using the same dosing regimen as the group receiving LHF-535 0.5 h prior to viral challenge.

*In vivo* efficacy of LHF-535 was evaluated using Tacaribe virus, an arenavirus closely related to Junín virus, but not known to be associated with significant human disease; this virus has been used to study arenavirus pathogenesis in AG129 mice [22]. Although AG129 mice lack a functional interferon response, this model is useful for early stage evaluation of antivirals that directly target the viral replication cycle. A daily oral dose of LHF-535 at 10 or 30 mg/kg protected mice from a lethal challenge with Tacaribe virus (Fig 3A) and dramatically reduced viral titers in plasma (Fig 3B), spleen (Fig 3C), and liver (Fig 3D). An increase in survival was also observed when the first dose of LHF-535 (10 mg/kg) was delayed by 1, 2, or 3 days after infection (Fig 4), demonstrating that LHF-535 is efficacious as a post-exposure therapeutic in mice.

### Development of LHF-535 resistance correlates with attenuation of virulence

**Fig 5:**
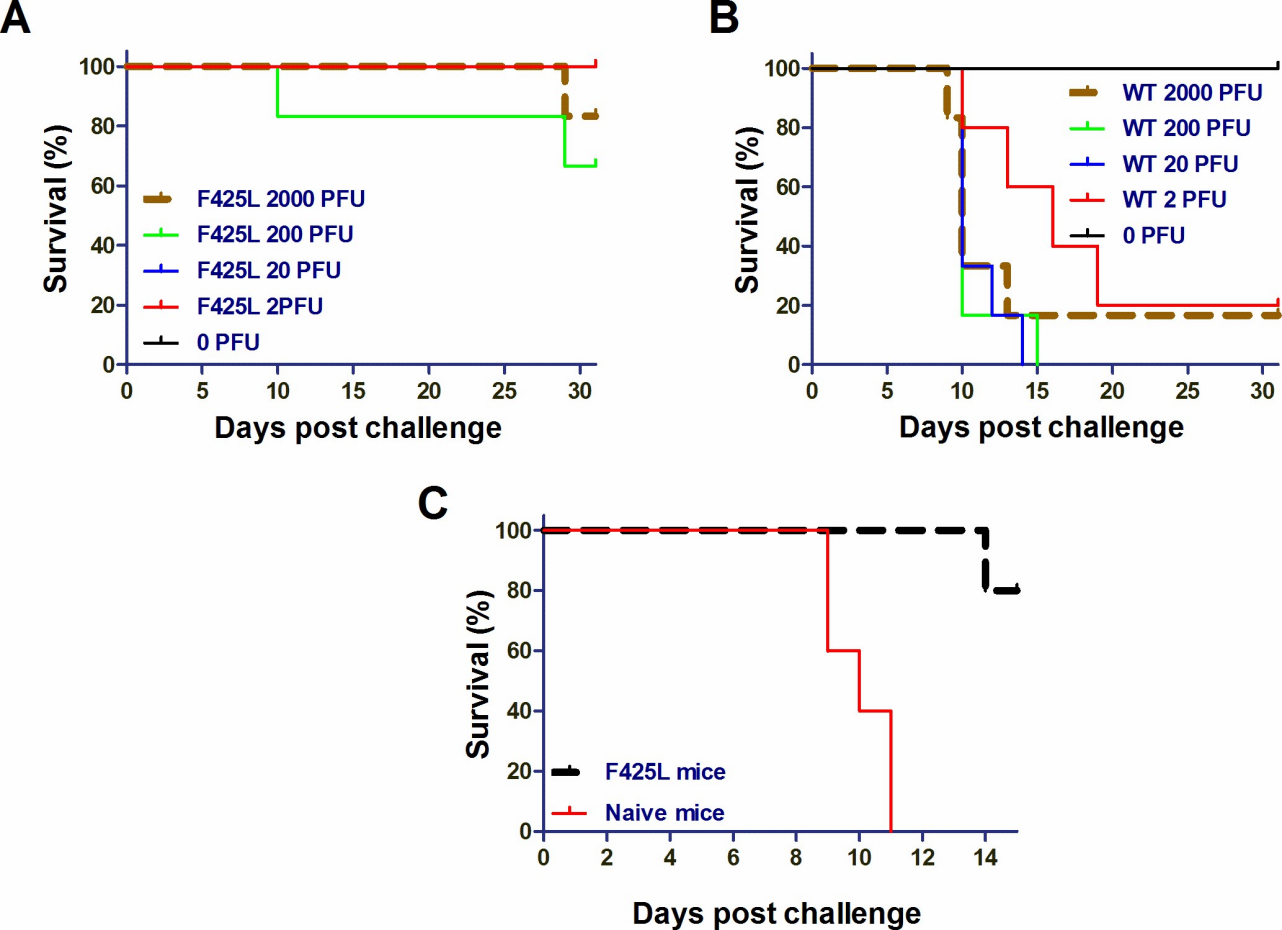
The LHF-535-resistant Tacaribe virus variant F425L is attenuated in AG129 mice and protects against subsequent challenge with the virulent wild-type virus. (A) LD_50_ study with F425L (*n* = 6 per group). (B) LD_50_ study with Tacaribe virus (*n* = 6 per group). (C) Mice (*n = 5*) infected with F425L (2,000 PFU) were challenged 31 days later with wild-type Tacaribe virus (200 PFU). Age-matched naïve mice (*n* = 6) were infected with wild-type Tacaribe virus (200 PFU) as a control.

Tacaribe virus has also been used as a surrogate system to study ST-193 sensitivity determinants [12]. Similarly, serial passage of Tacaribe virus in LHF-535-treated Vero cells was used to select virus isolates with reduced sensitivity to LHF-535; sequencing of these isolates identified amino acid substitutions in GP that were comparable to those observed following serial passage in the presence of ST-193 (Table 4). Interestingly, the majority of these isolates were attenuated in the AG129 mouse model (Table 4). One isolate in particular, F425L, contained an amino acid replacement at the same position as the Candid#1 attenuation determinant F427I. Tacaribe virus F425L was highly attenuated (Fig 5A) relative to the virulence of the parental virus (Fig 5B). Tacaribe virus F425L also functioned as an attenuated vaccine strain in this model, as mice previously challenged with this virus were protected from a subsequent challenge with the virulent parental Tacaribe virus (Fig 5C).

**Table 4:** LHF-535 sensitivity and pathogenicity of variants.

Isolate	IC_50_ (μM)	*In vivo* LD_50_
Wild type	0.001	< 2 PFU
A144V, V421A	> 0.1	< 2 PFU
F425L	> 5	> 2000 PFU
T434I	> 1	> 2000 PFU
F436L	> 5	> 2000 PFU
F436I	0.2	> 2000 PFU
A144V, C424G	0.05	6 PFU

## Discussion

LHF-535 potently inhibited viral entry mediated by GPs from all Lassa virus lineages and New World hemorrhagic fever arenaviruses evaluated in this study. Although the GPs from most Lassa virus strains exhibited sensitivity to LHF-535 that was essentially indistinguishable from one another, the GP from the LP strain was perceptibly less sensitive. The LP strain is the only reported member of lineage I and was the first isolated Lassa virus [23]. Lineage I is phylogenetically distinct from the other lineages and represents a basal position of the clade [9]. The LP strain is also distinct in that it is likely the product of sequential serial human passage. The first reported index case (L.W.) was a missionary nurse in Lassa, Nigeria; she died shortly after she was transported to Jos, Nigeria for evaluation [24]. The second case (C.S.), also fatal, was a nurse who had treated L.W. in Jos. The third case (L.P.) was Lily “Penny” Pinneo [25], a nurse who had treated both C.S. and L.W. Ms. Pinneo first developed symptoms 7 days after assisting at an autopsy on C.S. [24]. After Ms. Pinneo fell ill, she was evacuated to New York Presbyterian Hospital and ultimately recovered. The prototype Lassa strain LP was isolated from serum obtained from Ms. Pinneo 5 days after symptom onset [23]. It is likely that L.W., through her role as a nurse, acquired the disease from a patient. It is therefore possible that the LP isolate was the result of serial passage through as many as four or more individuals and the virus may have diverged from the initial zoonotic transmission.

Lassa virus isolates from Guinea, Liberia, and Sierra Leone are predominantly lineage IV viruses, whereas viruses of lineage II and III are found in Nigeria [9]. The absence of additional examples of lineage I viruses may be due to the remoteness of the source of the LP strain (northeastern Nigeria). It thus remains an open question whether the V434I substitution is a lineage I feature or is specific to the LP strain. Two related questions are whether the V434I substitution might have been selected during sequential passage in a non-natural host (human) and whether it might be linked to the L432I substitution (also unique to the LP strain) to compensate for fitness defects.

Recently, a new strain of Lassa virus was detected in Nigeria in a previously unrecognized host, the African wood mouse *Hylomyscus pamfi* [26]. This virus, Kako 428 (GenBank KT992425), has a distinct genetic profile, with the S segment (which encodes GP) clustering close to lineage I and the L segment clustering between lineages II and III. Kako 428 does not contain either of the transmembrane domain substitutions (L432I and V434I) found in LP, the prototype lineage I strain. It has been suggested that Kako 428 may be an evolutionary link between Lassa virus and related arenaviruses (e.g., Mobala and Mopeia viruses) that are not known to be associated with human disease [26]. On the basis of partial sequencing, four other Lassa virus sequences derived from *H*. *pamfi* samples also reportedly cluster with lineage I [26]; these partial sequences do not include the GP transmembrane domain. An additional new Lassa virus strain has recently been described and linked to Togo [27] and may represent a separate lineage; the GP sequence of this strain is conserved across all known LHF-535-sensitivity determinants.

Candid#1 is also less sensitive than Junín virus to arenavirus entry inhibitors other than LHF-535 [28], and F427I is implicated in this difference in sensitivity. While F427 has been shown to be a significant virulence determinant, it is likely that other loci are involved as well. For example, the Candid#1 parental strain XJ44 is attenuated in guinea pigs, but does not contain the F427I substitution [29]. Conversely, introduction of F427I into the virulent Romero strain of Junín was insufficient to completely eliminate viral dissemination and the development of mild clinical symptoms [21]. Machupo virus, the causative agent of Bolivian hemorrhagic fever, is attenuated *in vivo* by an F438I substitution in GP, analogous to F427I in Junín virus and V434I in Lassa virus [30]. While F427I is an attenuating substitution in the context of the Junín virus GP, the effect of the analogous V434I in the GP of the Lassa virus LP strain is unknown. The LP strain was isolated following a lethal outbreak, although from a patient that survived infection. Introduction of V434I into the Lassa virus GP has been proposed as a possible vaccine strategy; however, when this substitution is engineered into Lassa GP (along with flanking residues found in the Junín virus GP) it significantly reduces infectivity of lentiviral pseudotypes, reduces growth of recombinant Lassa virus *in vitro*, and exhibits genetic instability [17]. Interestingly, the Lassa LP strain produces only mild to moderate disease and no lethality in strain 13 guinea pigs [31]; in contrast, the Lassa Josiah strain generates uniform lethality in strain 13 guinea pigs, with an LD_50_ of less than 2 PFU [32].

The mechanism by which amino acid substitutions in GP can attenuate virulence is unknown. Viruses that are resistant to entry inhibitors do not generally show dramatic defects in fitness. Recombinant Romero strain F427I exhibits *in vitro* replication kinetics similar to that of the parental virus [21]. Although Candid#1 replicates less efficiently than Junín virus in mouse embryonic fibroblasts, both viruses replicate similarly in Vero cells [33]. The replication of Machupo virus F438I is similar to that of the wild-type virus *in vitro*, but Machupo virus F438I reverts to the wild-type sequence *in vivo*, in this case suggesting a cost to fitness [30]. Amino acid substitutions in GP can also affect the dependence of GP-mediated fusion on acidic pH. For example, Junín virus F427I is able to mediate membrane fusion at neutral pH, indicating destabilization of the pre-fusion complex [34]. Selection for mutations that destabilize the GP complex in the presence of LHF-535 is consistent with the proposed mechanism of action, which is stabilization of the GP complex and inhibition of the conformational rearrangement required for fusion [15]. Also supporting this mechanism of action is a recent report of an epistatic interaction between positions K33 of the Junín virus stable signal peptide (SSP) and F427 within GP2 [35]. Although K33 is highly conserved across mammalian arenaviruses, engineered mutations at this position modulate sensitivity to entry inhibitors [14], further implicating interactions between SSP and GP2 in the antiviral mechanism. Mutations that destabilize the pre-fusion complex could conceivably contribute to attenuation of virulence by increasing the proportion of defective particles or by allowing fusion to occur in a less regulated manner with respect to cell types or cellular compartments. In addition, Candid#1 exhibits greater dependence on human transferrin receptor 1 for mediating viral entry than does its virulent parental isolate [34]. Thus, it is also possible that the attenuation of virulence associated with resistance to entry inhibitors could be due to altered receptor specificity, thereby impacting the tissue or cellular tropism of the virus.

The emergence of drug-resistant viruses is an important aspect of antiviral therapy, so these results could have implications for the clinical use of LHF-535 or other arenavirus entry inhibitors. As Lassa fever is primarily a zoonotic disease, the likelihood of a drug-resistant strain emerging to replace the pathogenic, drug-sensitive strain appears low; however, person-to-person spread is reported, and nosocomial transmission is not infrequent [36]. Moreover, emerging drug-resistant strains are likely to be attenuated in virulence. Monitoring the emergence of drug-resistant viruses will be an important component of the clinical evaluation of LHF-535, and this study provides important context for interpretation. It will be useful to examine the sequence of clinical isolates, not only from patients that fail therapy, but also from those that recover, and to correlate outcome with any new sequence variants. These results also support the strategy of prophylactic co-administration of the Candid#1 vaccine with LHF-535 for cases such as accidental exposure to Junín virus, as LHF-535 interference with Candid#1 replication is expected to be minimal.

## Materials and methods

### Ethics statement

*In vivo* experiments were carried out in strict accordance with Kineta’s animal welfare policies (Public Health Service assurance #D16-00885), and recommendations in the Guide for the Care and Use of Laboratory Animals of the National Institutes of Health. The protocol, “#16–02 Small-molecule Antiviral Efficacy in Infection Models”, under which these experiments were conducted, was approved by Kineta’s Institutional Animal Care and Use Committee.

### LHF-535

Initial batches of LHF-535 were synthesized by Adesis, Inc. using a previously disclosed method [37]; subsequent batches were synthesized at PharmaCore, Inc. by the same method. For *in vitro* studies, LHF-535 was dissolved in dimethyl sulfoxide (DMSO) at a stock concentration of 10 mM. The compound was micronized at Powdersize, Inc. for use in *in vivo* studies.

### Cells and viruses

Vero and 293T cells were obtained from the American Type Culture Collection (ATCC; Manassas, VA). Vero cells were maintained in minimal essential medium (MEM) supplemented with 10% fetal bovine serum (HyClone Thermo Scientific, Logan, UT). 293T cells were maintained in Dulbecco’s modified Eagle medium (DMEM) supplemented with 10% fetal bovine serum, 2 mM L-glutamine, penicillin (100 U/ml), and streptomycin (100 μg/ml). Tacaribe virus strain TRVL 11573 was obtained from ATCC. The Candid#1 vaccine strain of Junín virus was provided by Robert Tesh (World Reference Center for Emerging Viruses and Arboviruses, The University of Texas Medical Branch, Galveston, TX). The Candid#1 virus stock (~10^8^ PFU/ml) was generated from a clarified lysate following one passage in African green monkey kidney cells (BS-C-1 from ATCC) and two passages in Vero cells. The molecular clone of the Romero strain of Junín virus was provided by Slobodan Paessler (University of Texas Medical Branch, Galveston, TX). The virus was rescued in baby hamster kidney fibroblasts (BHK-21 obtained from ATCC) and the stock (~10^8^ PFU/ml) was prepared from a single passage in Vero cells. Work with the pathogenic Romero strain of Junín virus was conducted in a BSL-3+ laboratory by vaccinated personnel.

### Tacaribe virus *in vivo* model

AG129 mice are IFN-α/β and–γ receptor-deficient mice. They were a kind gift from Michael Diamond (Washington University in St. Louis). For Tacaribe virus studies, we used mice that were sex- and age-matched (6–8 weeks old). All animal procedures were approved by the Institutional Animal Care and Use Committee (IACUC) and were conducted at Kineta in a BSL-2 facility. Experimental groups were sized (as specified in the figure legends) to allow for statistical analysis, and all animals were included in the analysis. All animal experiments were conducted in a non-blinded fashion.

For the LHF-535 dose titration study, mice were sorted into survival and titer arms and challenged by intraperitoneal (i.p.) injection with 200 PFU of Tacaribe virus. In the survival arm, mice were dosed orally with LHF-535 at 3, 10, or 30 mg/kg/day or with vehicle alone for 14 days with the first dose 30 min prior to infection. Micronized LHF-535 was suspended in 0.5% Methocel E15 and 1% Tween 80. The mice were observed for signs of morbidity and mortality. For the titer arm, mice were sacrificed at 7 days post-challenge; plasma, liver, and spleen samples were collected for assaying virus titers.

For the delayed treatment studies, AG129 mice were split into five groups with each receiving LHF-535 30 min prior, and 24, 48, and 72 h post infection along with a vehicle control group. All mice were challenged by i.p. injection with 200 PFU of Tacaribe virus and dosing ceased 14 days post-challenge. The mice were observed for signs of morbidity and mortality and were humanely removed from study if there were clinical observations of inactivity, labored breathing, or excessive weight loss (≥20%).

For the pathogenesis studies (LD_50_ determination), AG129 mice were infected with wild-type or mutant Tacaribe virus via i.p. injection using 10-fold serial dilutions of virus. The Reed-Muench method was used for LD_50_ calculations [38].

### Plaque assay

Tacaribe virus titers were assayed by plaque assay. Plasma and tissue homogenates were serially diluted and used to infect 12-well plates seeded with Vero cells (100,000 cells per well), and then incubated (37°C, 5% CO_2_) under 0.5% Avicel (RC-581NF, FMC Biopolymer), penicillin (100 U/ml), streptomycin (100 μg/ml), 1 mM sodium pyruvate, and 1 mM L-glutamine in a final volume of 0.5 ml per well. After 6 days, cell monolayers were fixed with 10% formalin (Azer Scientific) for 45 min, stained with 0.1% crystal violet in 20% methanol for 1 min, and scored.

### Pseudotyped virus production

Production of pseudovirions incorporating heterologous envelopes has been described previously [12]. GP genes were synthesized *de novo* (GenScript) and cloned into the mammalian expression vector pCAGGS [39]. The gene sequences not previously described [12] were Lassa LP (AF181853), Lassa AV (AF246121), Lassa 803213 (AF181854), Lassa GA391 (X52400), Lassa Weller (AY628206), Lassa CSF (AF333969), Lassa NL (AY179173), Lassa Z148 (AY628205), Lujo (FJ952384), LCMV Dandenong (EU136038), Chapare (EU260463), Mobala (AY342390), Gbagroube (GU830848), Menekre (GU830862), Mopeia (DQ328874), Flexal (AF512831), and Machupo Mallele (AY619645). Site-directed mutagenesis was performed at GenScript.

### Selection of LHF-535-resistant Tacaribe virus variants

Tacaribe virus variants with reduced sensitivity to LHF-535 were generated in four to five passages under selective pressure of increasing LHF-535 concentration (1–170-fold IC_50_) in Vero cells. Virus was harvested within 3–6 days and passaged again at a 5-fold higher concentration; in subsequent passages, this process was repeated two more times. After passaging, virus was plaque-purified, amplified in Vero cells, and evaluated for LHF-535 sensitivity. The GP genes of variants with altered LHF-535 sensitivity were sequenced.

### Antiviral assays

#### Junín virus yield-reduction assay

Virus yield reduction (VYR) experiments were conducted to determine sensitivity to LHF-535 in Junín Romero wild-type and vaccine strains. Varying concentrations of LHF-535 were added to test wells containing 70–80% confluent Vero cells just prior to infection at a multiplicity of infection (MOI) of approximately 0.002. Plates were incubated for 3 days, at which time virus-infected plates were frozen and thawed, and culture supernatants were collected for endpoint titration of infectious virus. The samples were plated on Vero cells and visual cytopathic effect was measured on day 10 post-infection. LHF-535 was tested in triplicate against both Candid#1 and the Romero strain. Work with the pathogenic Romero strain of Junín virus was conducted in a BSL-3+ laboratory by vaccinated personnel.

#### Tacaribe virus antiviral assay

Vero cells seeded in 96-well plates (5,000 cells per well) were infected with Tacaribe virus at an MOI of 0.1 following addition in triplicate of serial compound dilutions in DMSO. After 3 days, RNA was extracted from cell lysates (Promega SV 96 Total RNA Isolation System) for evaluation of Tacaribe virus RNA via qRT-PCR and the comparative *C*_T_ method [40]. Briefly, extracted RNA is used to generate cDNA with the High-Capacity RNA-to-cDNA kit (Thermo Fisher Scientific). For TaqMan based qPCR, reactions were performed with the cDNA using the TaqMan Fast Advanced Master Mix (Thermo Fisher Scientific) along with primers and a dual-labeled TaqMan probe set targeting a ~100 nucleotide region of GP (nt 809–912). 18S rRNA (VIC/MGB probe) from Thermo Fisher Scientific was used as internal control.

#### Pseudotype virus inhibition

293T cells were seeded in opaque 384-well plates (4,000 cells per well). The following day, LHF-535 (dissolved in DMSO) and DMSO alone were dispensed via an HP D300e Digital Dispenser to a final concentration of 0.2% DMSO in all wells. This was followed by addition of a fixed volume of lentiviral pseudovirions, 3 days incubation at 37°C, and measurement of luciferase activity (Promega Bright-Glo Luciferase Assay System). Test concentrations were performed in quadruplicate. Luminescence was averaged for each concentration or control (positive controls received DMSO alone, negative controls were mock-infected) and the 50% effective concentration was calculated using XLfit. Experiments were repeated multiple times to establish an average (geometric mean) EC_50_; experiments were repeated until the standard error of the mean across multiple experiments was less than one-quarter of the average.
